# Predictable changes in the accuracy of human papillomavirus tests after vaccination: review with implications for performance monitoring in cervical screening

**DOI:** 10.1038/s41416-024-02681-z

**Published:** 2024-04-13

**Authors:** Matejka Rebolj, Adam R. Brentnall, Kate Cuschieri

**Affiliations:** 1 Centre for Cancer Screening, Prevention, and Early Detection, Wolfson Institute of Population Health, Queen Mary University of London, London, UK; 2 Centre for Evaluation and Methods, Wolfson Institute of Population Health, Queen Mary University of London, London, UK; 3 Scottish HPV Reference Laboratory, Royal Infirmary of Edinburgh, NHS Lothian Scotland, Edinburgh, UK

**Keywords:** Cervical cancer, Cancer epidemiology

## Abstract

Vaccination against human papillomavirus (HPV) is changing the performance of cytology as a cervical screening test, but its effect on HPV testing is unclear. We review the effect of HPV16/18 vaccination on the epidemiology and the detection of HPV infections and high-grade cervical lesions (CIN2+) to evaluate the likely direction of changes in HPV test accuracy. The reduction in HPV16/18 infections and cross-protection against certain non-16/18 high-risk genotypes, most notably 31, 33, and/or 45, will likely increase the test’s specificity but decrease its positive predictive value (PPV) for CIN2+. Post-vaccination viral unmasking of non-16/18 genotypes due to fewer HPV16 co-infections might reduce the specificity and the PPV for CIN2+. Post-vaccination clinical unmasking exposing a higher frequency of CIN2+ related to non-16/18 high-risk genotypes is likely to increase the specificity and the PPV of HPV tests. The effect of HPV16/18 vaccination on HPV test sensitivity is difficult to predict based on these changes alone. Programmes relying on HPV detection for primary screening should monitor the frequency of false-positive and false-negative tests in vaccinated (younger) vs. unvaccinated (older) cohorts, to assess the outcomes and performance of their service.

## Background

Thirteen human papillomavirus (HPV) genotypes are considered carcinogenic or “probably” carcinogenic to humans: 16, 18, 31, 33, 35, 39, 45, 51, 52, 56, 58, 59, and 68 [1, 2]. All licensed vaccines contain valency for the two most oncogenic genotypes 16 and 18 [3] and have been shown to be highly efficacious. Population-based vaccination has achieved considerable success in many, but not all, parts of the world. Globally, coverage is associated with the countries’ income level, issues with vaccine hesitancy, COVID-related disruptions, and other factors [4–9]. Notwithstanding these important issues, several studies in vaccinated cohorts have demonstrated a reduction of anogenital HPV infections, cervical cytological abnormalities, all grades of cervical intraepithelial neoplasia (CIN) [10–14], and cervical cancer [15–18].

**Fig. 1:**
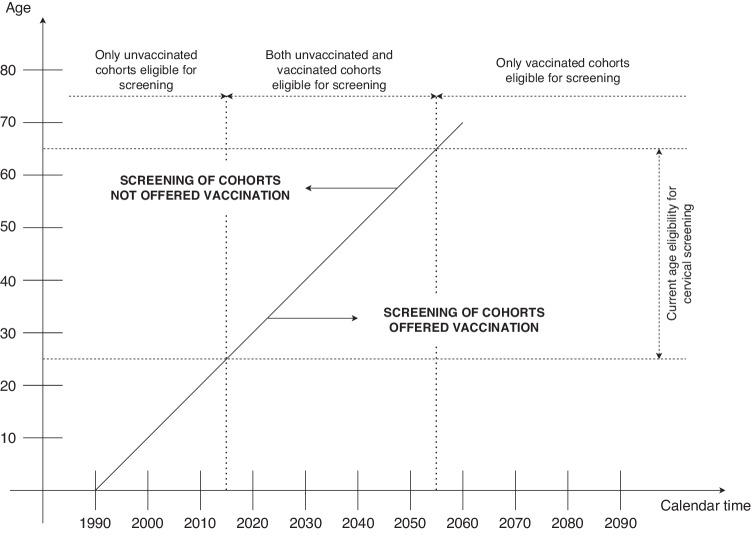
Gradual replacement of cohorts not offered HPV vaccination in a typical cervical screening programme. Screening eligibility in this example represents the current recommendations in the English programme, where screening is recommended between the ages of 25 and 64.

Vaccination is a key pillar of the global effort to eliminate cervical cancer, but population-based screening will need to continue for elimination to be achieved within the lifetime of today’s young girls [19, 20]. For decades to come, therefore, both vaccinated and unvaccinated women will be invited for screening (Fig. 1).

Owing to its high sensitivity for the detection of CIN2+ [21–23], HPV testing has become the international standard test for primary cervical screening [24–26]. HPV tests are designed to detect DNA or RNA viral target sequences of the 13 high-risk genotypes (and often also the “possibly” carcinogenic HPV66) [1]. A variety of different chemistries have been exploited in different assays to achieve this. Many are based on nucleic acid target amplification tests, although signal amplification approaches exist. Of the nucleic acid amplification tests, these can include broad-spectrum primers to cover the range of HPV genotypes, with genotype specificity conferred by specific probes, or genotype-specific approaches with multiple primer sets. Typically, HPV tests used for population-based screening have been calibrated to preferentially detect clinically relevant HPV infections associated with or pre-disposed to lesions [27]. Even then, the differences in the tests’ constitution and how they determine “clinically relevant” infections reveal themselves in discordant detection in the same woman; this has been consistently observed in multiple studies, particularly in samples without CIN2+ [28].

Two HPV assays, Hybrid Capture 2 (a commercial broad-spectrum signal amplification test; Qiagen, Hilden, Germany) and GP5+/6+ polymerase chain reaction test (an in-house broad-spectrum target amplification test), are considered “reference” tests by the community because their use was shown in randomised controlled trials (RCT) to detect progressive CIN2+ [21]. Having these reference tests has expedited the validation of new HPV assays by following an international consensus protocol [29]. In this protocol, HPV assay results are compared on aliquots from the same primary screening samples, collected fresh or retrieved from well-annotated biobanked collections [30, 31]. These so-called “Meijer criteria” use non-inferiority testing thresholds of the relative sensitivity and specificity for CIN2+ of a new test vs. a reference test. Best practice dictates that only tests validated according to these criteria should be used for population-based screening internationally and around 10 exist [32]. Other validation approaches with less strict sample inclusion criteria have also been proposed, but are not accepted as widely [33].

For vaccinated cohorts, one issue with continuing the current approach to test validation is that evidence for both reference tests (Hybrid Capture 2 and GP5+/6+) is from unvaccinated populations where HPV16/18 typically represent fewer than half of all infections but are over-represented in CIN2+ [34–38]. To date, and to our knowledge, no HPV tests have been explicitly validated as reference tests for vaccinated women.

As disease prevalence affects the subjective interpretation of cellular changes, the performance of cytology is unlikely to be the same in vaccinated and unvaccinated cohorts [39]. Likewise, we expect HPV test performance to change (see Box 1). In the rest of the paper, we review the recent literature on the effect of HPV16/18 vaccination on the epidemiology and detection of HPV infections and CIN2+ at the genotype level. Our goal was to further enhance our understanding of predictable changes in HPV test performance, with an aspiration to support screening programmes to develop their post-vaccination outcome and performance monitoring systems.

Box 1 Expected changes to HPV test accuracy in vaccinated populations In an unvaccinated population of size *N* that participates in HPV-based cervical screening, women can be classified depending on whether they have a prevalent CIN2+ lesion and their HPV test result in a 2×2 table:

**Table:** 

	CIN2+	<CIN2	Total
Test-positive	TP	FP	TP + FP
Test-negative	FN	TN	FN + TN
Total	TP + FN	FP + TN	*N* = TP + FP + FN + TN

Abbreviations: *CIN* cervical intraepithelial neoplasia. *FN*  absolute number of women with false-negative tests. *FP* absolute number of women with false-positive tests. *N* = total number of women screened with an HPV test, a sum of women with TP, FP, FN, and TN tests. *TN* absolute number of women with true-negative tests. *TP* absolute number of women with true-positive tests.

HPV16/18 vaccination has two notable direct effects on cervical cancer epidemiology: fewer women infected with high-risk HPV (leading to a decrease in test positivity TP + FP), and fewer women with CIN2+ lesions (a decrease in TP + FN). Thus, vaccination changes the relationship between TP, FP, FN, and TN. If we assume that (i) the number of women who test positive for HPV decreases to p*(TP + FP) and 0 < *p* < 1; (ii) the number of women with an underlying CIN2+ lesion decreases to q*(TP + FN) and 0 < *q* < 1; and (iii) the sensitivity of the HPV test (TP/(TP + FN)) remains unchanged; then in a vaccinated population we would expect the cells to change to:

**Table:** 

	CIN2+	<CIN2	Total
Test-positive	q*TP	p*FP + (*p*–*q*)*TP	p*(TP + FP)
Test-negative	q*FN	TN + (1–*q*)*FN + (1–*p*)*(TP + FP)	(FN + TN) + (1–*p*)*(TP + FP)
Total	q*(TP + FN)	(FP + TN) + (1–*q*)*(TP + FN)	*N* = TP + FP + FN + TN

Abbreviations: as above.

Then, the measures of HPV test accuracy for the detection of CIN2+ in unvaccinated populations and populations vaccinated against HPV16/18 are:

**Table:** 

	Unvaccinated population	Vaccinated population
Positive predictive value	$\frac{{TP}}{{TP}+{FP}}$	$\frac{q* {TP}}{p* ({TP}+{FP})}$
Specificity	$\frac{{TN}}{{FP}+{TN}}$	$\frac{{{{{{\rm{TN}}}}}}+(1-{{{{{\rm{q}}}}}})* {{{{{\rm{FN}}}}}}+(1-{{{{{\rm{p}}}}}})* ({{{{{\rm{TP}}}}}}+{{{{{\rm{FP}}}}}})}{{{{{{\rm{FP}}}}}}+{{{{{\rm{TN}}}}}}+(1-{{{{{\rm{q}}}}}})* ({{{{{\rm{TP}}}}}}+{{{{{\rm{FN}}}}}})}$
Sensitivity	$\frac{{TP}}{{TP}+{FN}}$	$\frac{q* {TP}}{q* ({TP}+{FN})}$

Abbreviations: as above.

This shows:

1. If the proportional vaccine-induced reduction in the burden of CIN2+ is larger than the proportional reduction in HPV infections (*q* < *p*), which is expected to hold with HPV16/18 vaccines, then the positive predictive value for CIN2+ in vaccinated populations will be lower than in unvaccinated populations. This has been observed in real-world data [14] and arises because non-HPV16/18 genotypes (not prevented by the vaccine) are less likely associated with CIN2+ than HPV16/18 [34].
2. In HPV16/18 vaccinated populations, HPV test specificity is unlikely to remain the same as in unvaccinated populations. In well-screened populations screened with highly sensitive but moderately specific HPV tests, the number of women with FN and TP tests is much less than the number of women with FP and TN tests. In this case, the formula above suggests that the specificity will be higher in vaccinated populations than in unvaccinated populations.

Note: the above analysis assumed that HPV test sensitivity does not change. However, it is unclear whether this assumption will hold. In particular, our review highlighted that HPV vaccination may lead to a lower viral load of infections in vaccinated women at screening. Therefore, estimates of test sensitivity in the vaccinated population based on distribution data from an unvaccinated population are at risk of bias due to spectrum effects [102, 103]. Viral load is a determinant of signal strength for an HPV assay, which is correlated with the likelihood that the infection will be detected [27, 51, 104, 105]. In other words, if a decrease in viral load occurs in vaccinated populations then the sensitivity of HPV testing may decrease compared with unvaccinated populations.

## Methodological approach

**Fig. 2:**
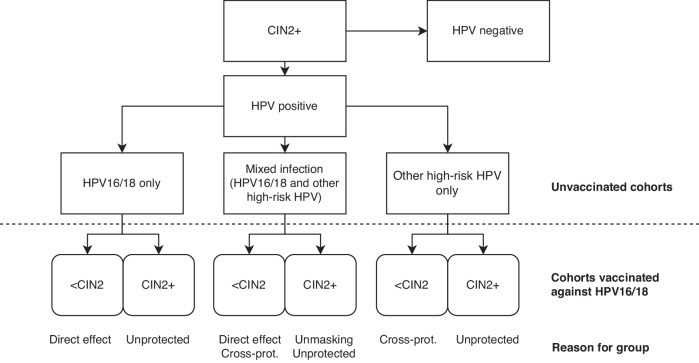
The potential effect of HPV16/18 vaccination on the prevalence of CIN2+ lesions, considering the direct effect on HPV16/18, cross-protection, and unmasking of other high-risk HPV genotypes. Note. The effects of the vaccine on the detection of CIN2+ are discussed in more detail in the paper. In short, direct effect: the intended effect of an HPV16/18 vaccine; cross-protection: an expected partial effect of an HPV16/18 vaccine on phylogenetically related non-16/18 genotypes; unmasking: an increase in the detection of non-16/18 high-risk genotypes due to fewer multiple infections involving HPV16/18 and therefore less competition for molecular resources within a test’s reaction (viral unmasking), or an increase in the detection of CIN2+ due to non-16/18 high-risk genotypes either because of better recognition of these genotypes as causal or because of fewer interruptions of lesion development once treatment of CIN2+ related to HPV16/18 no longer needs to take place (clinical unmasking). CIN cervical intraepithelial neoplasia. Cross-prot. cross-protection. HPV human papillomavirus.

To evaluate likely changes in HPV test accuracy due to vaccination, we consider the effect of the following vaccine-related phenomena on HPV test accuracy: direct protection against HPV16/18, herd protection, cross-protection, viral unmasking, and clinical unmasking. The effect of these phenomena on the prevalence of cervical disease (dichotomised as CIN2+ vs. <CIN2) is summarised in Fig. 2. To help consider mechanisms underlying each vaccine-related phenomenon, we discuss each in (hypothetical) isolation from the others. It is hard to quantify the effect sizes of these phenomena on test accuracy based on the available data on their own, or jointly, as relevant studies have been undertaken in diverse settings with a variety of inclusion criteria, HPV tests, and clinical pathways.

We assume that the protection afforded by the vaccine will be long-lasting [13, 40]. We focus here on vaccines against HPV16/18 because these vaccines were administered to young women who are offered cervical screening at present. In the last few years, several countries have switched to using the nonavalent Gardasil-9 vaccine (Merck, Darmstadt, Germany) [41, 42]. Gardasil-9 protects against five additional high-risk genotypes: 31, 33, 45, 52, and 58. Broader coverage of genotypes will likely change the strength of the vaccine-induced phenomena and their effect on the accuracy of HPV tests discussed below, compared to how these are expected to play out with bivalent and quadrivalent vaccines. As most countries vaccinate preadolescent girls and start screening at ages 25-30 years, nonavalent vaccines will start affecting screening outcomes from the end of this decade onwards. In the situation where screening eligibility for vaccinated cohorts is delayed to an older age group to maintain a cost-effective service [20], this may be moved even further into the future.

Our discussion considers HPV tests validated for primary screening regardless of additional triage tests. In screening programmes, triage tests increase the efficiency of the referral to colposcopy but do not affect the primary screening test results. This means that a negative triage test such as cytology does not reclassify a positive screening HPV test into a negative one, as HPV-positive/cytology-negative women are usually recommended for new tests in early recall with or without a colposcopy, instead of being directly returned to routine recall. An implicit assumption in our analysis is, however, that HPV-positive women would be triaged using the same protocols in unvaccinated and vaccinated populations.

**Fig. 3:**
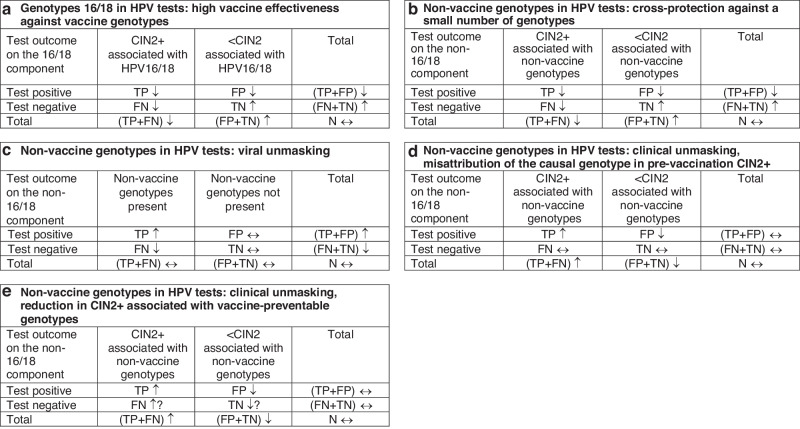
Expected direction of changes in true and false-positive and negative HPV tests following vaccination. Each mechanism is considered in the absence of other mechanisms. FN false-negative tests, FP false-positive tests, TN true-negative tests. TP true-positive tests. ↑=Likely to add to an increase for reasons given in the text. ↑?=Potentially adding to an increase but less certain for reasons given in the text. ↓=Likely to add to a decrease for reasons given in the text. ↓?=Potentially adding to a decrease but less certain for reasons given in the text. ↔=we have not identified reasons for a change.

To discuss potential performance changes of HPV tests, we make use of 2×2 tables based on the test result (HPV-positive vs. HPV-negative) and disease status (CIN2+ vs. <CIN2). The four cells in the tables are absolute counts of (1) those with disease, correctly identified (True Positives, TP), (2) those without disease, incorrectly identified (False Positives, FP), (3) those in whom disease was missed (False Negatives, FN), and (4) those without disease, correctly identified (True Negatives, TN; Fig. 3). Using 2×2 tables helps us consider the direct and indirect effects of vaccination on screening outcomes and provides insight into the likely direction of changes in the cells between a vaccinated and unvaccinated population. Related implications for the sensitivity (TP/(TP + FN)), specificity (TN/(FP + TN)), and the positive predictive value (TP/(TP + FP)) of an HPV test for the detection of CIN2+ are further discussed, but the negative predictive value (NPV, TN/(FN + TN)) is not considered separately. Because CIN2+ is uncommon and HPV testing is highly sensitive, NPV is close to 1. In the English HPV screening pilot that was undertaken in unvaccinated women, for example, the NPV for CIN3+ in three years after a negative HPV test was around 0.999 [22, 38], whereas in a multi-centre European study, it was 0.997 six years after a negative HPV test [43]. In vaccinated populations, the lower burden of lesions would drive the NPV even closer to 1, which is encouraging but less useful in practice for the purpose of identifying any issues with screening test accuracy.

## Anticipated vaccine-related changes to HPV test accuracy

### HPV16/18: direct protection in vaccinated women

HPV16/18 infections have been found in ~2-10% of well-screened unvaccinated women depending on the country, the starting age for screening (e.g., 20 vs. 30 years), and the HPV test [35, 44–46]. Data from RCTs consistently indicate that the vaccines are ~90% effective against 1-year persistence of HPV16/18 infections and ~90-100% effective against incident HPV16/18-associated CIN2+, in women without evidence of prior HPV exposure [47]. Similarly, real-world observational data indicate ~80–90% reductions in HPV16/18 infections and ~90% reductions in the associated CIN2+, particularly in women vaccinated before sexual debut [13, 14, 48, 49]. Additionally, it has been suggested that HPV16/18 infections in vaccinated women (i.e., breakthrough infections) may present with lower viral loads than in unvaccinated women [50], reducing both their likelihood of detection and progression to CIN [51–53]. Data from Kaiser Permanente showed that vaccination before age 18 years was associated with a halving of the three-year risk of CIN2+ after negative cytology at age 21–24 years [54]. Because of the relatively short follow-up [36], this is likely due to a reduction in HPV16/18 infections. Taken as a whole, these data support the hypothesis that vaccinated cohorts will have fewer FN screening tests due to the lower prevalence of disease post-vaccination (Table 1).

**Table 1:** A hypothetical example: expected changes in the rate of interval cervical cancer after a negative screening test before and after vaccination.

Population	Sensitivity for progressive CIN2+	Number of women	Expected number of cancers in 10 years without screening	Screening test outcomes	Cancers prevented through screening	Interval cancers (false-negative tests)	Interval cancer rate per 100,000 woman-years
Unvaccinated	95%	100,000	400	10,000 positive 90,000 negative	380	20	2.22 [20/(90,000 × 10)]
Vaccinated (70% effectiveness)	95%	100,000	120	5000 positive 95,000 negative	114	6	0.63 [6/(95,000 × 10)]
Vaccinated (70% effectiveness)	85%	100,000	120	5000 positive 95,000 negative	102	18	1.89 [18/(95,000 ×10)]
Vaccinated (90% effectiveness)	95%	100,000	40	5000 positive 95,000 negative	38	2	0.21 [2/(95,000 × 10)]
Vaccinated (90% effectiveness)	85%	100,000	40	5000 positive 95,000 negative	34	6	0.63 [6/(95,000 × 10)]

Assume that in the absence of any prevention (screening and vaccination) 400/100,000 women would develop cervical cancer in 10 years^a^ and that this risk decreases by ~70% [3] due to vaccination; that 10% of unvaccinated women test positive for HPV at screening and that this is reduced by ~50% [14] after vaccination; that HPV testing is ~95% [23] sensitive for progressive CIN2+ in unvaccinated women; and that loss to follow-up in 10 years is negligible. These calculations were repeated using an assumption that vaccine effectiveness for preventing progressive CIN2+ was 90%.

Note. The numbers in this table should not be interpreted as representative for any contemporary screening programme. This is because (1) the background risks of cervical cancer in screened women (i.e., the incidence that would have been observed had there been no screening and no vaccination) are changing [100] and are difficult to estimate reliably for discrete timepoints, and (2) the size of the vaccine effect on the risk of cervical cancer across the age span, and including cases irrespective of the causal genotypes, is as yet uncertain (thought the effect on cancers that affect young women, likely dominated by HPV16/18 [38, 72], has been shown to be substantial) [15–18].

^a^Based on data from Denmark, where it was estimated that ~3% of women developed cervical cancer sometime during their life (15-85 years) before screening was introduced in the 1960s [80]. In a population of 100,000, therefore, 3000 cases would be expected to be diagnosed across 70 years of life, or around 400 every 10 years on average. In the tabulated example, it was assumed for simplicity that all women undergo screening (both populations), and that vaccination protects women across the entire age span (vaccinated population).

In vaccinated cohorts, direct protection will lower the absolute numbers of women with positive HPV tests (TP + FP) and CIN2+ (TP + FN) that are due to HPV16/18 (Figs. 2 and 3a). Further, because vaccine effectiveness against HPV16/18 is so high, it is likely that all three categories: TP, FP, and FN will be affected so that their absolute numbers will decrease, and that, in a population with a fixed size N, these decreases will be compensated by an increase in the absolute number of women with TN tests. This will lead to an increase in specificity of the HPV16/18 test component as the PPV decreases (Table 2). The number of breakthrough HPV16/18 infections will likely be small, so the increase in specificity will likely have a greater impact on the efficiency of screening programmes compared to a reduction in the PPV. Although following the real-world data referenced above the expectation is that the absolute number of FN tests will decrease, it is unclear whether the relationship between the numbers of TP and FN will be affected. Hence, changes to the sensitivity of the HPV16/18 test component are more difficult to predict. A change in the average viral loads post-vaccination affecting the ability of HPV assays to detect clinically relevant infections could lead to lower test sensitivity, but this is at present less well understood and would need to be confirmed in future studies.

**Table 2:** Summary of predictable changes in HPV test accuracy due to HPV16/18 vaccination, described in detail in Fig. 3 and in text in the section ‘Anticipated vaccine-related changes to HPV test accuracy’.

HPV genotypes	Vaccine-related phenomenon	Sensitivity	Specificity	PPV
HPV16/18	Direct effect on HPV16/18 infections in vaccinated women (section ‘HPV16/18: direct protection in vaccinated women’, Fig. 3a)	↔	↑	↓
	Herd immunity for HPV16/18 infections in unvaccinated women (section ‘HPV16/18: herd protection in unvaccinated women’)	↔	↑	↓
Non-vaccine high-risk HPV genotypes	Cross-protection against non-vaccine high-risk genotypes phylogenetically related to HPV16/18 (section ‘Non-vaccine high-risk genotypes: cross-protection’, Fig. 3b)	↔	↑	↓
	Viral unmasking of non-vaccine high-risk genotypes in multiple infections due to reduced competition for molecular resources in an assay (section ‘Non-vaccine high-risk genotypes: *viral* unmasking’, Fig. 3c)	[↑]^a^ ↔	[↔]^a^ ↓?	[↑]^a^ ↓?
	Clinical unmasking of non-vaccine high-risk genotypes due to misattribution of the causal genotype to HPV16/18 in pre-vaccination CIN2+ cases (section ‘Non-vaccine high-risk genotypes: *clinical* unmasking due to misattribution of the causal genotype in pre-vaccination CIN2+’, Fig. 3d)	↑?	↑?	↑?
	Clinical unmasking of non-vaccine high-risk genotypes due to a reduction in the cervical treatment of CIN2+ associated with HPV16/18 (section ‘Non-vaccine high-risk genotypes: *clinical* unmasking due to a reduction in vaccine genotype CIN2+’, Fig. 3e)	↔	↑?	↑

All changes are considered to take place in vaccinated women except herd immunity, which manifests itself in unvaccinated women. All changes refer to clinical measures of test accuracy (against the detection of histological endpoints such as CIN2+), unless otherwise specified. Notes. ↑=Likely to add to an increase for reasons given in the text. ↑?=Potentially adding to an increase but less certain for reasons given in the text. ↓=Likely to add to a decrease for reasons given in the text. ↓?=Potentially adding to a decrease but less certain for reasons given in the text. ↔=We have not identified reasons for a change, though may be uncertain for reasons given in the text.

^a^Analytical accuracy, measured against virological endpoints (detection of non-16/18 HPV genotypes).

### HPV16/18: herd protection in unvaccinated women

Owing to an overlap of sexual networks between vaccinated and unvaccinated women, partial herd protection against HPV16/18 has been observed in unvaccinated women, particularly in settings with a high vaccination coverage [10, 13, 14, 55, 56]. Some of the above considerations in section ‘HPV16/18: direct protection in vaccinated women’ relating to vaccinated women may therefore also apply to unvaccinated women. These indirect vaccine-induced changes might affect the accuracy of the HPV16/18 test component in unvaccinated women in a similar way as direct changes in vaccinated women, but likely to a lesser degree.

### Non-vaccine high-risk genotypes: cross-protection

Vaccines against HPV16/18 may also partially protect against other phylogenetically related genotypes. In the PATRICIA RCT evaluating a bivalent vaccine against HPV16/18 (Cervarix; GSK, Brentford, UK), the protection against persistent infections with genotypes 31 and 45 was estimated at ~78% (77.1%, 95% CI: 67.2-84.4, and 79.0%, 95% CI: 61.3-89.4, respectively), and that for genotype 33 at ~43% (43.1%, 95% CI: 19.3-60.2) [47]. In the FUTURE RCT evaluating a quadrivalent vaccine against HPV6/11/16/18 (Gardasil; Merck, Darmstadt, Germany), protection against a persistent infection with HPV31 was estimated at ~46% (46.1%, 95% CI: 15.3–66.4) [47]. Observational studies have also reported a cross-protective effect, particularly for HPV31 [13, 57, 58]. Furthermore, sparse data have suggested that vaccination might also reduce viral loads of non-vaccine genotype infections compared with those in unvaccinated cohorts [50], which might indicate a reduced likelihood of detection and risk of progression to CIN2 +  [53]. Among English women aged 24–25 years, for example, the reduction in CIN2+ associated with 12 non-vaccine genotypes in combination—likely due to cross-protection against some of these—was estimated at ~30% for women who were vaccinated at age 14–17 [14]. The cross-protective effect tends to be weaker when women receive fewer than three vaccine doses and may wane over time even with a full three-dose schedule [47, 57].

The likely effect of cross-protection on test accuracy is shown in Fig. 3b. This effect is expected to decrease the absolute number of women with positive HPV tests (TP + FP) and CIN2+ (TP + FN) associated with non-vaccine high-risk genotypes. It is likely that the absolute numbers of both FP and TP tests would decrease and, in a population of fixed size N, be compensated for by an increase in the absolute number of TN tests. The latter is expected to increase the specificity for the detection of CIN2+ of the non-vaccine genotype test component. If the contention that vaccination decreases viral loads of non-vaccine genotypes is real, then this is likely to reduce the PPV for CIN2+. With fewer CIN2+ left to be detected, it is also likely that the absolute numbers of FN tests would decrease. As above (section ‘HPV16/18: direct protection in vaccinated women’), however, it is unclear whether this would affect test sensitivity (Table 1).

### Non-vaccine high-risk genotypes: *viral* unmasking

Despite cross-protection working to decrease the prevalence of non-16/18 genotypes, some real-world data studies have actually reported an increase [13, 55, 58]. For example, a meta-analysis of studies in women younger than 20 years found a statistically significant doubling in the detection of HPV52 and HPV56 after bivalent vaccination, and a statistically significant 20-30% increase in the detection of HPV39, HPV51, and HPV59 after quadrivalent vaccination [58]. Considering type replacement to be unlikely for HPV infections [59, 60], a potential driver of this observation is viral unmasking. Viral unmasking occurs in infections with multiple genotypes after eradication of HPV16/18 reduces the competition for molecular resources within a test’s reaction—allowing amplification/detection of other genotypes [13, 58, 61–64]. The phenomenon is more likely in populations with high pre-vaccination levels of HPV16 [62] and with HPV tests that rely on a consensus rather than a genotype-level detection approach. There is substantial scope for viral unmasking because multiple infections are common [60, 65–68].

As illustrated in Fig. 3c, the viral unmasking phenomenon would not change the absolute number of women with disease associated with non-vaccine genotypes, defined here as “true” (rather than detected) infections (TP + FN), or its complement (FP + TN). It is expected to increase the absolute number of TP tests (they are now “unmasked” and reported as detected on an HPV assay) and, because TP + FN remains fixed, decrease the absolute number of FN tests. Viral unmasking is expected to contribute to the increased total number of women with positive tests (detected infections; TP + FP). Consequently, it is likely to increase *analytical* sensitivity and *analytical* PPV (i.e., the sensitivity and the PPV for the detection of non-vaccine HPV genotypes rather than histologically confirmed CIN2+). With the total number of women without true infections (FP + TN) remaining stable, it is not clear that viral unmasking should affect the relationship between the absolute numbers of women with FP and TN test results, so the phenomenon would likely not affect *analytical* specificity.

Changes in analytical accuracy due to unmasking might translate into changes in clinical accuracy. As non-vaccine genotypes are less likely to cause CIN2+ than HPV16/18 [34, 36], higher analytical sensitivity may manifest itself, in the non-vaccine genotype test component, as a decrease in clinical specificity and the PPV for CIN2+ (Table 2). Clinical sensitivity of HPV testing overall (regardless of genotype), however, may be less affected. This is because the detection of a single high-risk genotype is usually enough to trigger clinical follow-up. This way, a previous “failure” to detect a causal but masked non-vaccine genotype in unvaccinated women would have been inconsequential clinically.

### Non-vaccine high-risk genotypes: *clinical* unmasking due to misattribution of the causal genotype in pre-vaccination CIN2+

HPV16/18 infections are the fastest and most likely genotypes to progress to cervical cancer [3]. Consequently, CIN2+ diagnoses detected in routine screening are often automatically considered a consequence of an HPV16/18 infection if those genotypes were found in the preceding tests, with or without any co-infections. Once HPV16/18 infections are reduced through vaccination, however, one may record more non-16/18 CIN2+ than previously due to potential hierarchical misattribution. This form of clinical unmasking has been examined in several studies. After a hierarchical attribution based on genotyping preceding cervical samples, a study including 276 women with CIN2+ observed that 67% of the lesions were “caused” by HPV16/18 [69]. However, after further sophisticated microdissection of lesions and genotyping, the proportion attributable to HPV16/18 reduced to 52% (*p* < 0.0001), whereas the attributable proportions for other genotypes, particularly 35, increased [69]. Similar patterns were observed in another study [70]. Other evidence supporting this effect includes a microsimulation modelling study calibrated to co-infection data from England [71]. The study suggested that once HPV16/18 are eradicated and any remaining lesions are attributed correctly to non-vaccine genotypes, the incidence of CIN2/3 would appear to be 5-7% higher than expected based on masked data, and that of cervical cancer 4–5% higher.

Likely effects of these changes on the HPV test are summarised in Fig. 3d. In a population with a fixed size N, we expect it to increase the absolute number of women with TP non-16/18 HPV tests, increase the absolute number of cases with CIN2+ reported to be associated with non-16/18 genotypes (TP + FN), and decrease its complement (FP + TN). The total absolute numbers of women with positive tests (TP + FP) and negative tests (FN + TN) would likely not be affected with this mechanism; hence, the absolute number of women with FP tests would decrease but it is unclear whether the absolute numbers of FN and TN tests would change. Under these circumstances, the sensitivity of the non-vaccine high-risk genotype component of an HPV test would likely increase, as would the PPV and the specificity. Change in specificity, however, is expected to be small if the number of CIN2+ cases misattributed to HPV16/18 in an unvaccinated population is small.

### Non-vaccine high-risk genotypes: *clinical* unmasking due to a reduction in vaccine-genotype CIN2+

CIN related to HPV16/18 tends to develop faster and at an earlier age [36, 38, 72], but can be prevented through vaccination; this also prevents any related excisional treatments. It has been hypothesised that the absence of treatment at an earlier age may, over time, unmask lesions arising from non-HPV16/18 infections that would have co-infected the cervix but were then removed jointly with the treatment of HPV16/18-related lesions, or would have infected the cervix after that treatment but be prevented from progressing because the tissue that is critical to lesion development had been removed [73, 74]. Data from the Costa Rica Cervarix RCT provide some support for this phenomenon. Here, by year 11 the reduction in CIN2+ associated with any high-risk genotype was 27.0/1000 (57.5/1000 in vaccinated vs. 84.5/1000 in unvaccinated women) [73]. This overall reduction was composed of two effects: a reduction of 36.2/1000 in CIN2+ due to vaccine-preventable genotypes (defined as 16/18/31/33/45), which became apparent at the beginning of the follow-up, and an increase of 9.2/1000 due to other high-risk genotypes, which only became apparent towards the end of the follow-up [73]. Given the ability of the transformational zone to (partially) regenerate after excisional treatment [75–77], however, future studies that quantify the extent of this phenomenon in routinely vaccinated populations would be of value.

Likely effects of this form of clinical unmasking are summarised in Fig. 3e. In a population of fixed size N, we expect that the absolute number of women with CIN2+ (TP + FN) would increase, meaning that the absolute number of women without CIN2+ (FP + TN) would have to decrease. When this mechanism enhances the progression of non-vaccine infections to CIN2+ but does not change the risk of an infection (i.e., unchanged absolute numbers of women with positive (TP + FP) and negative tests (FN + TN) with more TP tests and fewer FP tests in Fig. 3e), the PPV would increase. If any increase in the absolute number of women with FN tests is small compared to the decrease in the absolute number of women with FP tests, and consequently the decrease in the absolute number of women with TN tests is also small (from a relatively large baseline), then the specificity may nominally increase. Changes in the sensitivity are difficult to predict.

## Validation of HPV tests for vaccinated populations

Our analysis suggests that organised screening programmes should expect to see a variation in HPV test accuracy for vaccinated and unvaccinated cohorts, even when the same HPV test is used for both. Further complexity is expected because (a) vaccine effectiveness and its effect on test accuracy depend on the age of vaccination and the number of administered doses, but both have varied over time and between countries; and (b) some of the effect of the vaccine on test accuracy may be imminent whereas other changes may only manifest gradually or intermittently.

**Fig. 4:**
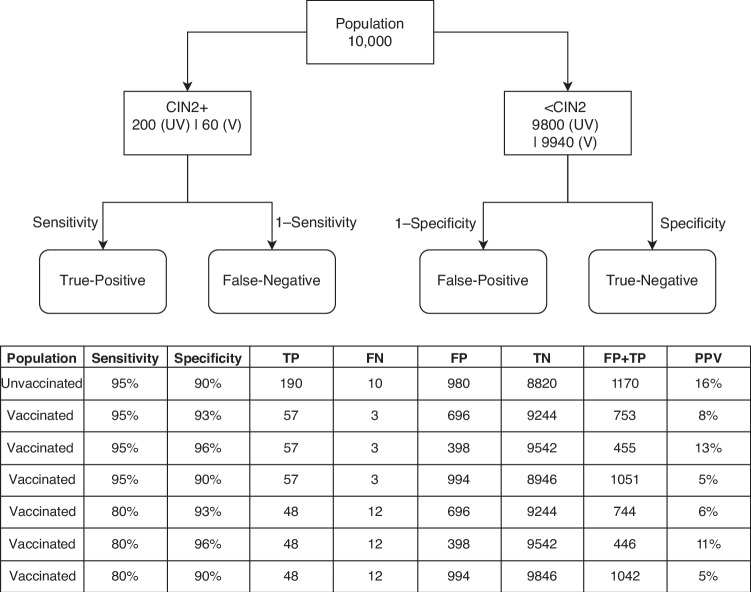
Changes in the distributions of absolute numbers of screened women with true-positive, false-negative, false-positive, and true-negative tests, the total number of women with positive tests (requiring referral to early recall and/or colposcopy), and the positive predictive value for CIN2+, depending on the changes in HPV test sensitivity and specificity after vaccination. The total size of the screened population is 10,000. In an unvaccinated population, 2% are assumed to have CIN2+ [101]. The effectiveness of the vaccine is assumed to be 70% [3]. In an unvaccinated population, the sensitivity of HPV testing is assumed to be 95% [23] and the specificity 90% [43]. While in unvaccinated women HPV16/18 infections represent about a third of all HPV infections [38], they are decimated in vaccinated populations. Therefore, in a vaccinated population the base scenario uses a specificity of ~93%. CIN cervical intraepithelial neoplasia, FN false-negative tests, FP false-positive tests, PPV positive predictive value for CIN2+, TN true-negative tests, TP true-positive tests, UV unvaccinated women, V vaccinated women.

This calls for more work to be dedicated to explaining vaccine-induced changes in the accuracy of HPV tests because uncertainties such as those that we discussed here represent a challenge for the ability of screening teams to interpret the observed outcomes and quality assure their processes [78]. The hypothetical example based on a plausible set of parameters in Fig. 4 further helps to quantify the effect of these uncertainties on the planning of the capacity of the screening service. Although vaccination has the potential to increase test specificity, the PPV for CIN2+ would remain low. Even relatively small changes in test specificity would substantially affect the numbers of women who require further clinical management and a referral (either to early recall or to colposcopy, or both). An improvement in test specificity, which is the more likely direction of change, could lead to idle referral capacity; whereas even a relatively small decrease may, despite vaccination, require similar referral capacity as in an unvaccinated population. This uncertainty in the required capacity volumes could lead to serious difficulties in providing an uninterrupted screening service [79]. Furthermore, any reduction in test sensitivity could increase the number of missed cases of CIN2+ and increase their chances of progression to cancer. While this is not a surprising observation, it is important to note that unresolved decreases in test sensitivity could substantially diminish the extra advantage in reducing the residual risk of cancer that women derive from vaccination on top of the advantage that they derive from screening alone.

With these potential consequences in mind, there might therefore be a case for validation studies in vaccinated cohorts of already licenced HPV tests. These are not yet feasible, however, as defining a reference test to a similar standard in vaccinated as in unvaccinated populations will require more work.

Another issue related to the validation of HPV tests for vaccinated populations is the definition of the target condition. In unvaccinated populations, this has usually been CIN2+ or CIN3+ because treatment of CIN2/3 is instrumental in preventing the progression to cancer [21, 80, 81]. Non-vaccine genotypes are, however, less likely to cause cancer even once they have already caused CIN2/3 [34]. Hence, vaccination will weaken the association between CIN treatment and cancer prevention and thereby change the character of an “average” CIN2/3 case detected through screening. To a degree, the uncertainty regarding the endpoint for test validation may be diminished by focusing on CIN3+ rather than CIN2+, as CIN3 is more likely to progress to cancer [82]. However, this approach does not address women with progressive CIN2. A more inclusive approach to improve the classification of CIN2+ related to non-16/18 genotypes could rely on the development and validation of ancillary approaches using biomarkers indicative of progression such as p16, various immune-related markers, methylation of specific genome regions, and other genetic alterations as predictive of progressive CIN [83–88]. However, most of these studies so far have been small and, because they were undertaken in unvaccinated populations, may have been driven by CIN caused by HPV16/18. Further validation of biomarkers focusing specifically on lesions related to non-HPV16/18 genotypes would be beneficial.

## Pragmatic approaches to monitoring the accuracy of HPV-based screening

A pragmatic approach to understanding test accuracy in vaccinated cohorts is to monitor the numbers of FN and FP outcomes for HPV tests currently in use in screening programmes. Such monitoring would help services identify potential issues with their tests. An analogous area where the utility of this approach has been demonstrated is HPV self-sampling. Here, monitoring with real-world data in some settings has suggested an increased number of FN tests and the need for optimisation of sample processing protocols [89, 90]. Though not meant to replace well-designed validation studies, monitoring vaccinated and unvaccinated cohorts separately, and comparing performance, would provide useful information regarding FN and FP tests. In some countries, vaccinated women have been screened for years with the same HPV-based protocols as unvaccinated women [22, 91]. Pooling of these data from several settings to increase numbers may also be helpful, as was the case with evaluating the reference Hybrid Capture 2 and GP5+/6+ tests [21].

FN tests from observational data are often determined based on interval cancer incidence, where interval cancers are defined as those preceded by (false-)negative screening tests [21, 92–95]. This approach is challenging for vaccinated women, particularly in smaller populations, as the accumulation of the cases required for a robust analysis may take years. Another caveat to monitoring based on interval cancers is that their rates are expected to decrease when the background risk of cancer decreases, as well as when there are moderate but true decreases in a test’s sensitivity for progressive CIN. Thus, changes in test performance may be hard to detect particularly when the true effectiveness of the vaccine is uncertain (Table 1). An alternative to using interval cancer incidence is to compare outcomes from two consecutive screening rounds and measure the frequency of CIN3+ detected at round 2 subsequent to a negative HPV test in round 1 [22]. There are benefits to using CIN3+ as a proxy for cervical cancer: unlike cancer, CIN3 develops soon after an infection [36, 96] and will be more common than interval cancer.

To aid recognition of issues with test sensitivity, screening services could define the maximum expected thresholds of the measured condition and their acceptable variation (confidence intervals). As a starting point, a threshold could be based on a reduction of cancers, or indeed CIN3+, by ~70% in line with the attributable fraction of HPV16/18 infections globally [3]. This could be refined with country-specific data or any new evidence on the effectiveness of the vaccines. Note that special provisions may need to be made for truly HPV-negative cancers [97, 98]. These are unlikely to be affected by vaccination and may become over-represented in post-vaccination assessments of interval cancers even if their number remains unchanged.

FP cervical screening tests are usually defined as positive screening tests that are not followed by histologically confirmed CIN2+ at direct or early recall colposcopy referral. The FP test frequency could be compared between the vaccinated and unvaccinated populations after accounting for the expected reductions due to vaccination. As an example, consider the unvaccinated population undergoing HPV-based screening in England. Here, 5–27% of the women tested positive for HPV depending on their age, and around one-third of these infections included HPV16/18. CIN2+ was detected in 0.5–6% of screened women, again with a strong age gradient and with around one-half of the cases containing HPV16/18 [38]. Assuming 100% effectiveness of the vaccine for HPV16/18 and not taking viral unmasking into account, the frequency of FP tests should then decrease by 15–30%.

## Conclusions

In summary, HPV test performance in vaccinated cohorts is likely to differ from that in unvaccinated cohorts due to the direct protective effect against HPV16/18; and cross-protection, viral unmasking, and clinical unmasking affecting the epidemiology of non-16/18 high-risk HPV genotypes. We outlined the likely directions of the effects of these on test accuracy, but the overall implication remains unclear due to a lack of direct evidence. Development of HPV test evaluation and validation frameworks in vaccinated cohorts would help collect such evidence and is therefore urgently needed. Until such guidelines are available, monitoring of screening outcomes in vaccinated and unvaccinated cohorts separately could help understand changes in test accuracy and contribute to the development of new reference standard(s). For this, IT infrastructures that allow for a linkage between women’s vaccination status and their screening outcome records will be crucial [99].

Beyond this, the field could also consider prioritising the development and evaluation of new cervical screening tests that are agnostic to the HPV genotype. The current HPV tests are extremely effective in reducing the burden of cervical cancer, but they require early recall testing which can stretch across several years and may result in up to one in 10 screened women having to cope with a FP outcome. Hence, both vaccinated and unvaccinated women would benefit from new cervical screening tests that would prioritise the detection of markers of abnormal cell transformation. It is likely that the value of such tests would increase with the nonavalent vaccine.

## Data Availability

The review was based entirely on previously published data.
